# Institute of hydraulic engineering and water resources management (RWTH Aachen University): an overview of research focus and training

**DOI:** 10.1186/s12302-018-0146-0

**Published:** 2018-06-01

**Authors:** Elisa Classen, Holger Schüttrumpf

**Affiliations:** Institute of Hydraulic Engineering and Water Resources Management, RWTH Aachen University, Mies-van-der-Rohe-Strasse 17, 52074 Aachen, Germany

**Keywords:** Engineering, Flooding, Coasts, Sediments, Hydraulic energy, Hydrodynamics, Ethohydraulics, Morphodynamics

## Abstract

Water is an essential element and highly valuable resource in life. Between the priorities of environment, people and economy, it is of increasing importance to fully understand the fundamental force of water to be capable of handling waterborne events—such as flooding—manage and ensure water quality and availability, and utilize hydraulic energy. The Institute of Hydraulic Engineering and Water Resources Management (IWW) at RWTH Aachen University has a long research tradition in this field. Going back to the founding year of the university in 1870, the chair is based on the work of civil engineer Otto Intze, who is best known for his pioneering contributions in construction of dams and elevated water tanks. Ever since then, the institute has broadened its research spectrum and is today focusing on flood protection structures, hydraulic engineering design, integrated coastal zone management, morphodynamics and ethohydraulics. In a comprehensive approach, physical model experiments are combined with field measurements and numerical simulations to investigate a wide range of projects. With its annually organized International Symposium on Hydraulic Engineering (IWASA), the institute also offers information to a wide audience on highly topical aspects in the field of water engineering works and water management, while at the same time bridging the gap between science and industry. The institute is part of the “Project House Water”, a research network at RWTH Aachen University that was established within the framework of the German excellence initiative. Here, scientific competencies from the fields of ecotoxicology, process engineering, geography, sociology, economy and hydraulic engineering are focussed to allow for an interdisciplinary, holistic assessment of flooding events and their impacts.

## Background

### About 150 years of hydraulic engineering in Aachen—beginning and development of the IWW

**Fig. 1:**
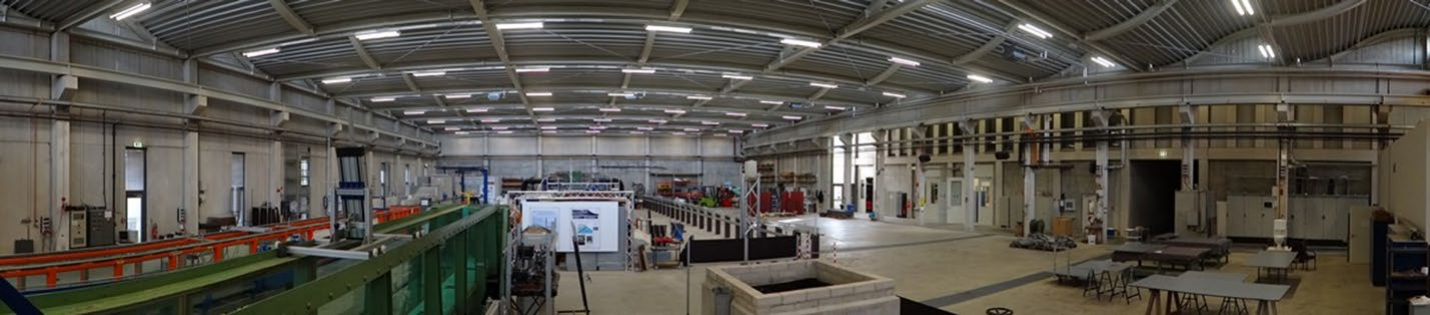
Experimental hall at IWW

In the late nineteenth century, civil engineer Otto Intze started the long tradition of engineering at RWTH Aachen University—at that time still called the *First Prussian Institute of Technology*: With his first lectures in hydraulic engineering, Intze prepared the ground for fundamental and applied research in the field of water engineering, flood control and dam construction in Aachen. The name of the Institute of Hydraulic Engineering and Water Resources Management (IWW) and its professoriate changed in the following decades, but the research focus was always kept. In 2013, the chair was provided with a new office building and experimental laboratory hall: a floor area of 2250 m^2^ housing permanently installed tilting, stepped and annular flumes and a water circulation system with flow rates of up to 1500 l/min and a volume of 400 m^3^ provide excellent opportunities for large-scale model experiments. Apart from that, the hall is equipped with a modular 30 m flow channel, used for ethohydraulic studies, overflow and dike experiments. The open space can be used for temporary installations and smaller physical model experiments (Fig. 1). The Institute operates an own model building department and is assisted by metrologists to support the individual research projects in-house and during fieldwork; as of beginning 2017, 43 scientific and non-scientific staff members are employed.

The institute is associated with a non-profit friends association to support teaching and research activities. The association also provides assistance in funding conference attendances, lectures and field trips and financially contributes to the publication of scientific work. To promote the contact between academia and industry, the International Symposium on Hydraulic Engineering, Aachen (IWASA) is held every January, for almost 50 years. It is renowned among experts and gives the opportunity to discuss highly topical water themes as well as to establish contacts for future ventures.

## Main text

### Research in the field of hydraulic engineering at IWW

The research area of the IWW is broadly based and includes key topics from the fields of high water risk management, hydraulic engineering structures, navigable waterway constructions, hydroelectric power, pumped-storage plants, fish protection elements, sediment transport and morphodynamics, coastal engineering and water quality improvement. Projects are typically assessed both in fundamental and applied research, to not just give an understanding of the involved processes but recommend options for tailor-made solutions. Within its projects, the institute is working in close cooperation with research facilities and administrative boards in Germany and worldwide, such as the Federal Waterways Engineering and Research Institute (BAW), the German Federal Institute of Hydrology (BfG), the Ministry for Climate Protection, Environment, Agriculture, Conservation and Consumer Protection of the State of NRW (MKULNV) or the Israel Institute of Technology. The IWW is further integrated into the Project House Water [1], a joint venture of six RWTH institutes with a research focus on water. The project house is funded by the Exploratory Research Space (ERS) at RWTH Aachen University and aims to promote interdisciplinary projects under one roof by combining expertise from different faculties. Including, but not limited to the project house, the following research fields are currently in focus of the institute IWW.

### Flood risk management

High water events recurrently pose an existential threat to life and property of individuals, infrastructure, agriculture and industry in flood-prone regions. Within minimal time, personal and cultural assets can be damaged or irretrievably destroyed during a flood disaster. These events have lasting effects for human and nature and echo for a long time, such as the disastrous flooding alongside Elbe and Danube in 2013 or the flooding of the Oder river in 2010.

Yet, flooding risks are not limited to inland waters but also occur at open waters as a result of weather phenomena such as Hurricane Matthew in the Western Atlantic (2016) or the tsunami in Japan in 2011, which also led to a nuclear disaster at the Fukushima plant. While the private, non-material loss can hardly be quantified, flooding events cause substantial costs amounting to billions, such as hurricane Katrina in the US (2005) which killed more than 1800 people and caused damage of over 80 billion US$. These numbers clearly emphasize the importance for the development of flood protection and flood management tools. Due to human intervention, like river regulations and land sealing, as well as an increasing dense settlement in flood-prone regions, the need for research in this field is also underlined.

**Fig. 2:**
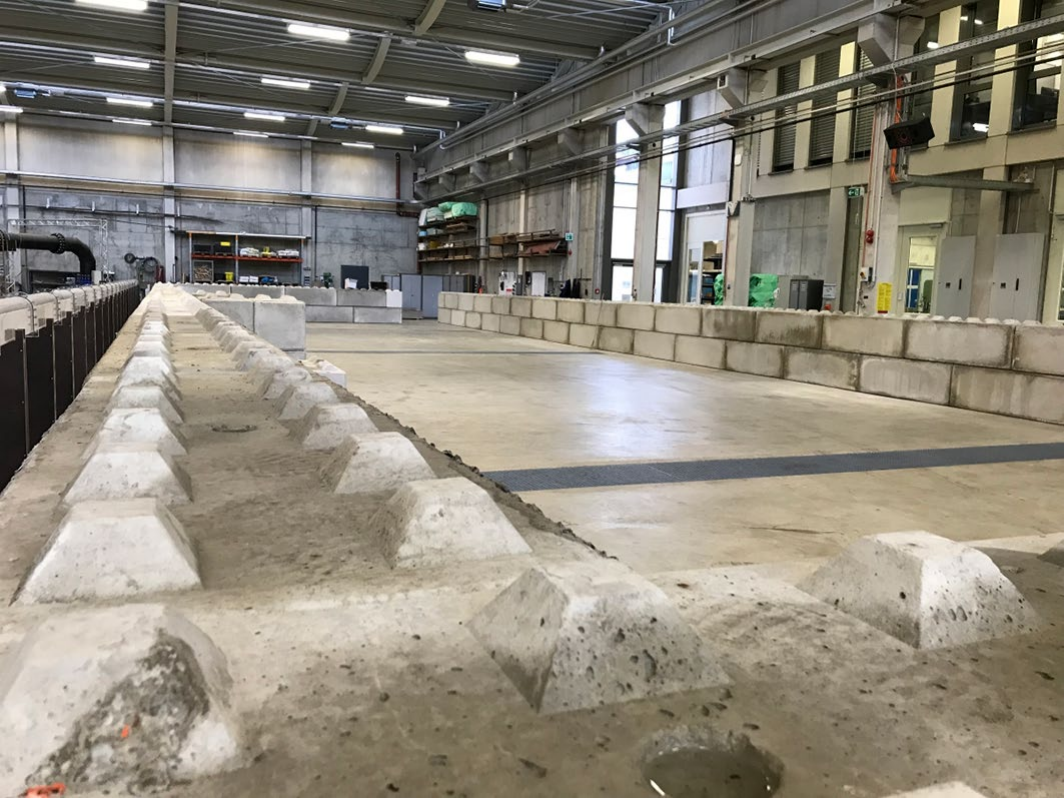
Construction work on a physical model of Havel polder. A base frame with a length of 34 m and width of 11 m is set up with concrete building blocks to form a basin; afterwards, a model dike with a breach will be constructed inside. Flooding this structure will give insight into the filling characteristics, so that constructional measures for an intended filling of the polder can be concluded

For about 20 years, high water risk management is a core topic at IWW: as an important element in flood control is the technical protection by construction measures, IWW is evaluating the physical processes of flooding to identify weak points, which lead to structural breakdowns upon flood events. Through this, suggestions for improvement of existing and new flood defence systems are defined [2]. For instance, flood polders are used to improve water retention and thus attenuate flood peaks in the course of small-scale and average flood events. Breach formation processes and their influencing factors are still subject to a number of uncertainties. Within the BfG commissioned project “Physical Model Tests of Dike Breaches” within the framework of the “National Flood Protection Program for Optimization of Havel Polder”, the targeted filling of flood polders through dike breaches is assessed and technically optimized. In that context, physical model tests of dike breaches will be performed in the hydraulic laboratory at IWW to quantify the influence of geometric, hydraulic and geotechnical boundary conditions on dike breach formation (Fig. 2).

Further current projects at IWW address issues related to revetment stability during swell load (project HYGEDE, supported by BMBF). In addition, coupling mechanisms of high and ground water are analysed [3] to minimize flood damage in underground buildings such as tunnels and car parks.

**Fig. 3:**
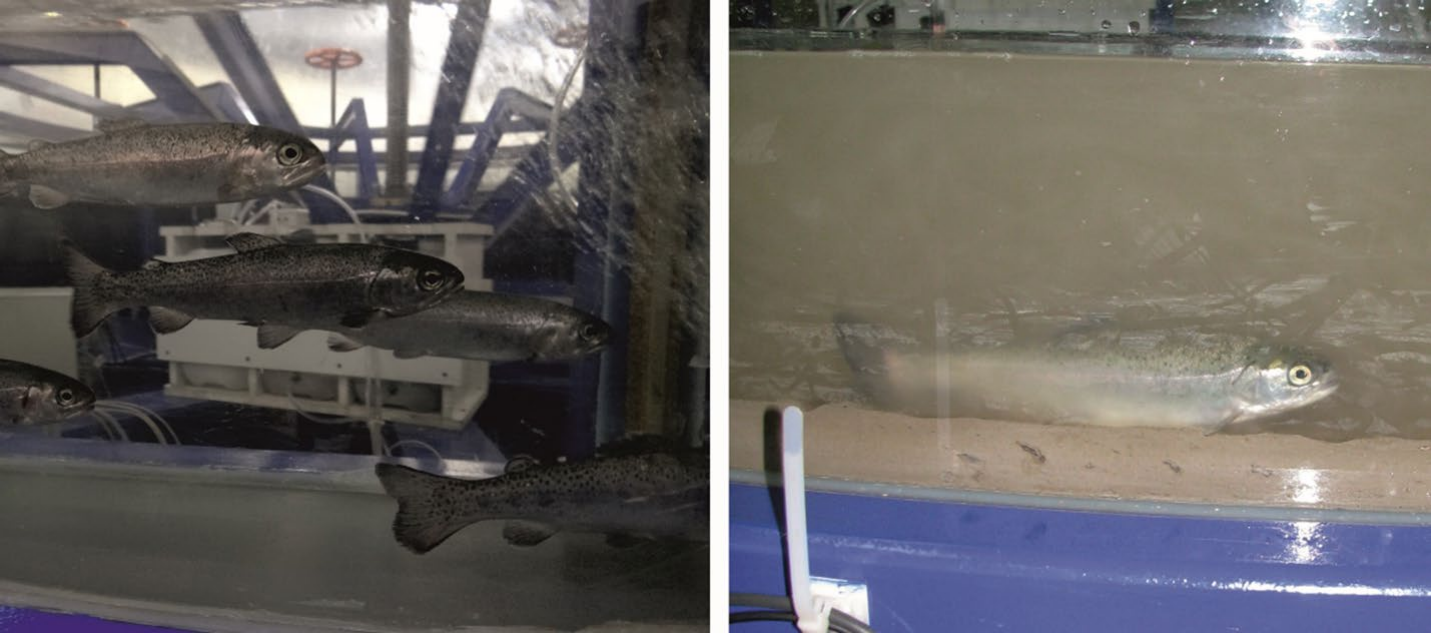
Hydro-toxic studies on sediment-bound pollutants in the circular flume at IWW. Rainbow trouts (*Oncorhynchus mykiss*) were exposed to clear water (negative control, left) and water–sediment-mixtures (right) that were artificially spiked with environmental pollutants, e.g., polycyclic aromatic hydrocarbons

While the aforementioned topics relate to the immediate effects of flooding, a consequential problem originates from pollutants (soluble as well as sediment associated) that are spread during high waters and which can impair entire ecosystems. Whereas the hazardous potential of chemicals is mostly known, the long-term effects of so-called emerging pollutants (e.g., pharmaceuticals, personal care products, biocides) is not fully understood yet and, for this reason, is investigated at IWW in interdisciplinary projects [4, 5] (Fig. 3).

In general, it is observed that high water models and surveys from past decades have to be adapted to take into account the ongoing influence of the global climate change. In consequence, related recommendations towards flood risk management have to be critically reviewed [6].

### Hydraulic engineering design

**Fig. 4:**
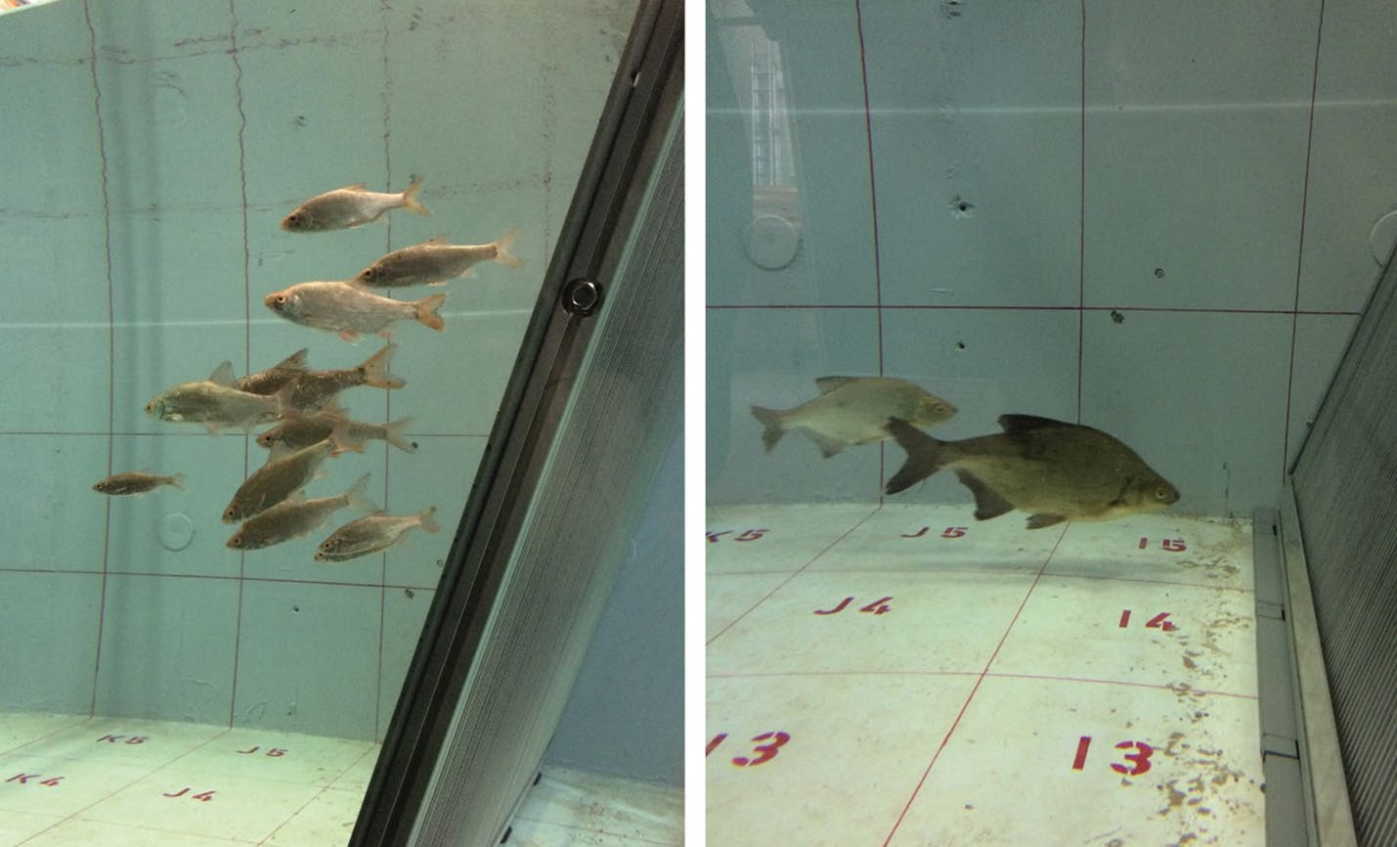
Behavioural studies on fish within the flow channel. Different types of bar racks are tested on common roaches (*Rutilus rutilus*, left) and common breams (*Abramis brama*, right)

Hydraulic structures cover a variety of waterside buildings that can basically be categorized into two groups: the first set comprises constructions with a protective function, like retention reservoirs, dikes, bank reinforcement structures or flood barriers. The second group includes constructions that enable the personal and industrial use of water, such as drinking water networks, irrigation facilities, waterways, wastewater treatment systems or hydropower stations. Against the background of the European Water Framework Directive of 2000, which aims to ensure a good chemical and ecological quality of water bodies, hydraulic engineering is set in a sense between conflicting priorities, namely the requirements of engineering itself and those of sustainability and environmental protection. In accordance with the directive, alternative fish protection structures up- and downstream of water facilities are developed and optimized to allow for a safe fish passage. In this context, studies are performed at IWW, to analyse the orientation and search behaviour of fish in front of bar racks that are typically used to prevent flotsam and animals from entering pipes and turbines (Fig. 4).

By understanding the movement habits of fish at such barriers, alternative solutions can be engineered to improve animal welfare without significant loss in value for the affected companies.

**Fig. 5:**
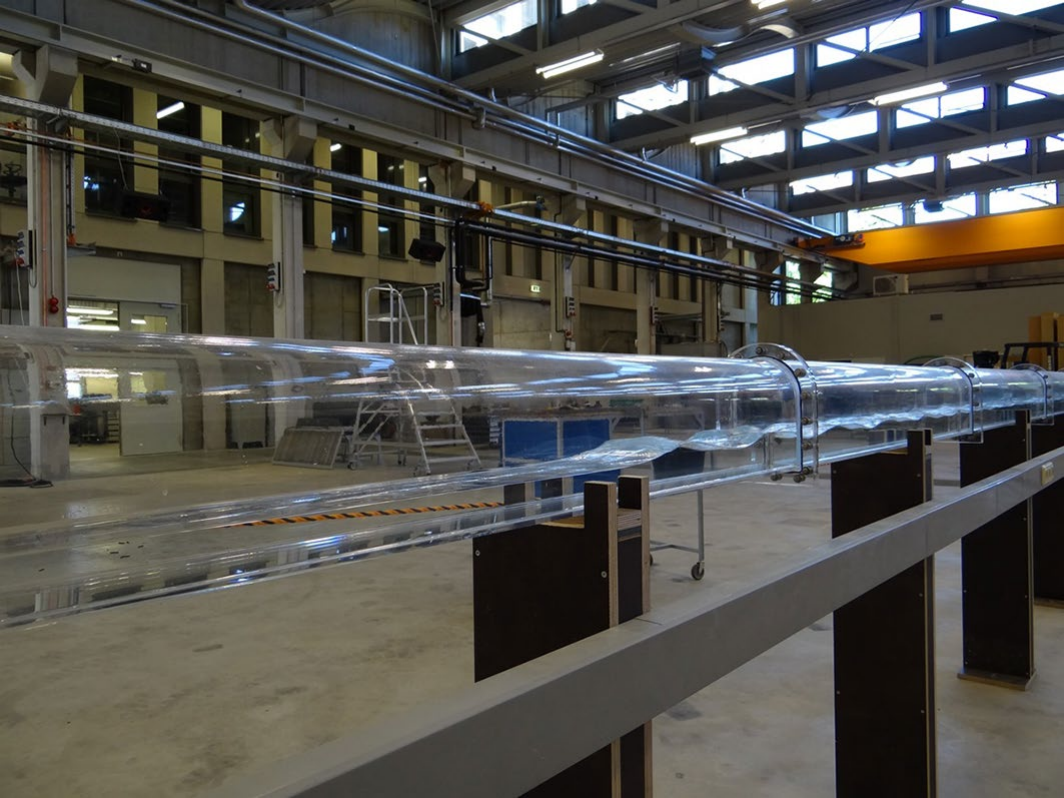
Model of underground pumped-storage plant. With a length of about 36 m, the model is used to assess flow conditions in cavities

The combination of hydraulic engineering and environmental sustainability is approached in further projects at IWW: with phasing out nuclear energy in Germany until 2022, alternative energy sources have to be exploited. An overall challenge with renewable energies is their volatility: for instance, solar power is limited by day/night cycles and wind farms are dependent on weather conditions. Therefore, an important aspect in promoting alternative energy concepts is the development of reliable energy storage systems. Overground pumped-storage plants have been used for this purpose for over 100 years: in a first step, electricity is used to pump water to a higher elevated reservoir. Once, energy is needed, the water is released to a lower basin using the difference in altitude to drive generators. As this operating mode requires special topological conditions (large-scale basins at different heights), the construction of such plants has not only a high environmental impact but is limited to mountainous landscapes. To overcome these restrictions, pumped-storage plants should be developed to work underground using for example existing caverns from former ore or coal mining. Thus, the main disadvantages of overground pump storage reservoirs (topography, nature conservation and often large distances between place of energy production and place over energy consumption) can be bridged. A major challenge of this new type of pump storage plant is the filling and emptying process of the underground caverns to ensure energy storage and energy production in a given time interval. Therefore, researchers from IWW numerically and experimentally analyse hydrodynamic processes during filling and draining of cavities, to define requirements for the underground reservoirs (Fig. 5). These specifications can be used either to characterize existing caverns according to their usability or to create completely new underground storage plants. Nowadays, the development of underground pumped-storage plants is in a very early stage. Several ideas were developed but no underground pump storage reservoir was realized so far. This is due to the uncertainty of the geological conditions, the unknown processes during filling and emptying including interaction with the geological properties, operation and placement of turbines and pumps in several hundreds of meter depth below the surface and other reasons. Anyway, the idea of placing a pump storage reservoir in the underground is promising and implementation might be possible after more research is carried out and open gaps are solved.

### Coastal engineering

**Fig. 6:**
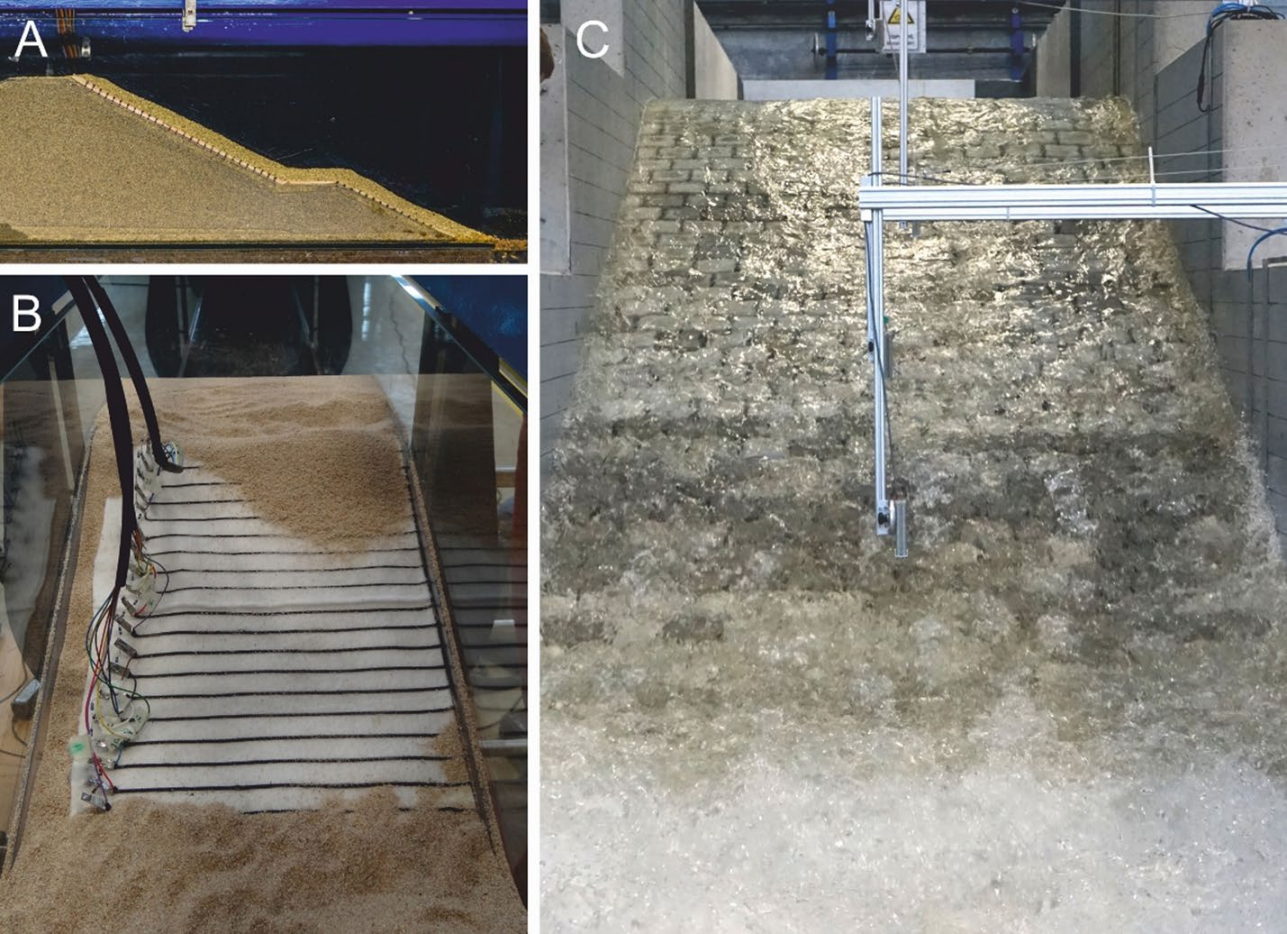
Dike models used in project EarlyDike. **A** Small-scale model, equipped with intelligent geotextile (**B**) for detection of water; **C** overflow of large-scale dike model

Coastal regions have always had an attraction to people and business: some of the most influential cities reside at waterside, where they represent gateways to the world; enormous values have accumulated there in the past centuries. Moreover, coastal habitats also pose a high ecological value as they substantially contribute to the overall ecosystem services (up to 40%) [7]. Yet, these landscapes are endangered; about 20% of the European coastline is seriously affected by erosion. In addition, frequency and intensity of storm surges have increased in the past while at the same time a rise of sea-level by up to 80 cm is predicted for this century due to global warming [8]. Protection of coastal habitats has, therefore, become a fundamental future challenge: in terms of engineering, it is important to understand static loads and mechanical stresses on hydraulic structures upon extreme water-related events. At IWW, this topic is addressed, for example, in the BMBF-funded project EarlyDike, which focuses on the development of early warning systems to detect sea dike failures at a very early stage: at present, dike monitoring typically includes annual inspection of viewable dike areas, such as controlling height and cover condition to detect any weak points (e.g., signs of erosion, changes in vegetation, burrowing animals). Existing warning systems, however, only take into account observed and forecasted changes in water level; there is no system yet to monitor structural properties inside sea dikes. This is the starting point for project EarlyDike, which uses sensors-equipped geotextiles to detect water intrusion inside dikes (Fig. 6).

Yarn-like carbon sensor fibres are attached to a geotextile in parallel strands and the fabric is then placed between the sand core and the clay layer of a dike, facing landwards. With two types of sensor fibres, this setup allows for monitoring humidity and structural stability of the dike: if water is intruding the structure, it will be signalled by a change in conductivity between two parallel sensor strands. A strain of the fabric, as caused by deformation of the dike, can also be detected through the specific sensor fibres. Both parameters are captured real-time and allow for quick protective and action measures in case of an impending dike failure [9]. The studies at IWW are supported by numerical analysis and aim to develop a flooding simulation software to predict flooding-affected areas, resulting consequences and mechanical durability of protective structures.

**Fig. 7:**
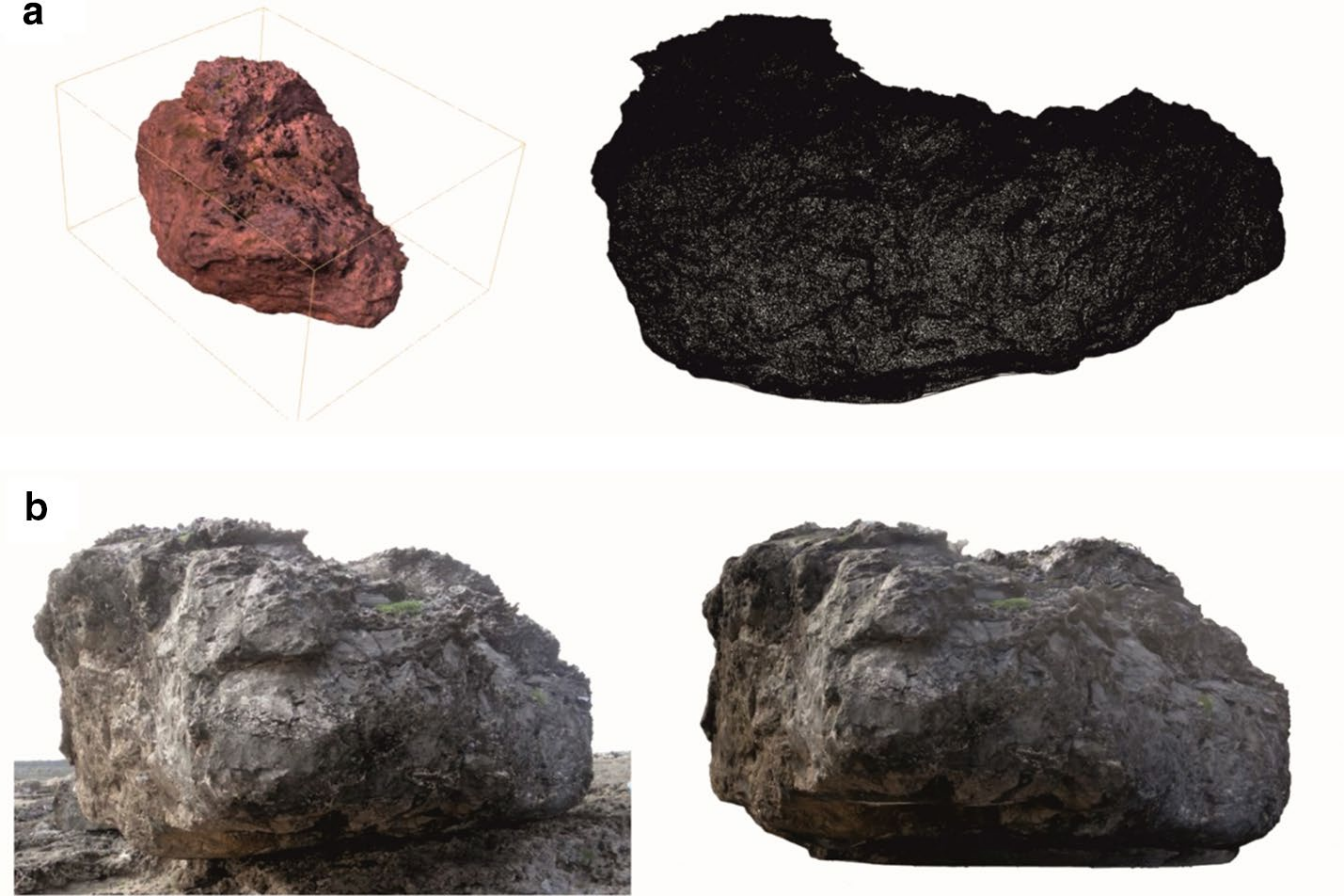
Analyses on boulders. **a** Perspective view and mesh model of a boulder deposited on the Island of Bonaire, Lesser Antilles, Caribbean Sea; **b** comparison between the original photograph and the numerical model

Another extreme water-related incident that affects coastlines are tsunamis, which originate from storms or seaquakes. Due to their infrequent, but sudden occurrence, the data situation is limited and it is rather difficult to determine physical parameters from recent events. For this reason, researchers at IWW take a look back in history: large boulders have been found close and distant to coastal regions, covered by marine sediments, indicating that they originate from open waters. These rocks and their position in relation to the sea are used in a numerical, inverse approach to calculate forces necessary to move the boulders to the mainland (Fig. 7). Results are validated by model experiments and can be used afterwards as basis for designing protective structures at coastlines.

### Morphodynamics

**Fig. 8:**
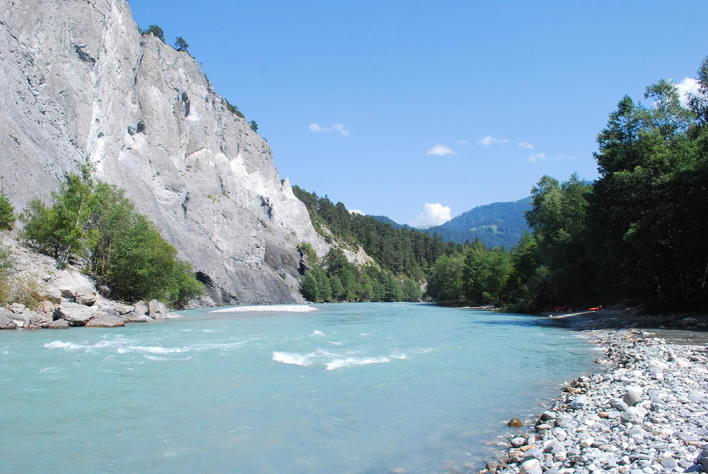
Anterior Rhine in the Ruinaulta in canton Grisons, Switzerland

River systems are dynamic structures, shaped by movement of water and sediments; the riverbed is thereby subject to a continuous accumulation and erosion of geological and biological materials. The field of morphodynamics focuses on these changes in river structure and sediment fluxes on various timescales, ranging between minutes and centuries. Here, the morphology of rivers is affected not only by natural factors, such as a rise in precipitation levels but also ecological processes and anthropogenic interventions can have major influences (e.g., glacial melting due to global warming). The steady growth of waterway transport increases the demand for improved inland waterways and ports, resulting in substantial changes of river morphology due to dredging activities at riverbeds, straightening of watercourses and building of hydraulic structures. In a comprehensive approach, covering the complete river course from spring to estuary, the sediment balance of the river Rhine was analysed over a period of 20 years as a collaborative work of IWW and BfG [10]. It showed that the Rhine can be divided into four sections, namely the alpine, impounded, free-flowing and delta section, each of which displays characteristic morphodynamics and sediment fluxes (Fig. 8). Sources and sinks of different sediment types were identified and balanced, taking into account data from previous studies using echo soundings and sediment transport data.

Gathered information from the Federal Waterways and Shipping Administration as well as the federal provinces has been merged with own data collected at IWW. This includes, for example, measurements to assess the transport of fine and medium sand, as the bed-load collectors used in the included studies have a mesh size larger than those particles. To estimate the amount of sand that was not captured in the previous studies, bed-load collectors identical in construction were installed in the tilted flume at IWW and flushed with water and a defined sediment mixture, representative of the Rhine bed. Comparing the dry weights of the sediments accumulated in the collector and those initially applied revealed average sand losses between 10 and 50% depending on the type of collector used and the composition of the bed-load sample. Based on these results, a correction factor was determined to allow for adjusting previously acquired data to display the actual amount of transported sand.

As an overall result, the sediment balance study of the river Rhine offers a baseline for future research on the river Rhine as well as concepts for technical and constructional measures, including sediment management.

While the mere relocation of sand, gravel or clay is a visible sign of sediment flux, an associated issue is not noticeable at first sight: as noted before, sediments are often contaminated with harmful substances arising from pharmaceuticals, chemically loaded wastewaters or fertilisers. By movement of these sediments, the contaminants are further spread and constitute an ecological hazard. This last aspect is leading back to the research field of flood risk management presented at the very beginning of this article and nicely demonstrates the cross-linking of research projects at IWW.

### Teaching

The Institute of Hydraulic Engineering and Water Resources Management is a member of the faculty of civil engineering at RWTH Aachen University. It offers lectures and seminars as part of the study programs civil engineering, business administration and engineering, environmental engineering, and mobility and transport. The institute also provides interdisciplinary and cross-faculty teaching in the fields of ecotoxicology, wastewater management and georesources management. The range of courses includes not only basic subjects such as hydromechanics, hydrology and hydropower, but also allows specialization in the disciplines of navigable waterway construction, water management and surface mining, and risk management. The course portfolio is expanded by domestic and international field trips, internships at engineering consultancies and in the construction industry and gives the opportunity to work on topical research projects at the institute. With its experimental hall and its teaching equipment—e.g., a mobile, modular laboratory system to demonstrate operating modes of hydraulic tubes and turbines—IWW allows students to study hydraulic engineering not only from a fundamental perspective but also from an applied point of view.

## Conclusion

The importance of water-focussed research is expected to further increase in the next years: changes in climate conditions as well as population growth will provoke an active confrontation with these topics to not only ensure availability and quality of drinking water but also to protect nature and human beings from water-borne events. Resource-preserving action is also required to protect water bodies on one hand and to foster a rethinking in terms of energy production on the other hand. The work of hydraulic engineering institutes—such as IWW—will contribute to these challenges by investigating related processes and developing effective tools and measures.

## Abbreviations

BAW: Bundesanstalt für Wasserbau (Federal Waterways Engineering and Research Institute)
BfG: Bundesanstalt für Gewässerkunde (The German Federal Institute of Hydrology)
BMBF: Bundesministeriums für Bildung und Forschung (Federal Ministry of Education and Research)
BWK: Bund der Ingenieure für Wasserwirtschaft, Abfallwirtschaft und Kulturbau e.V
DFG: Deutsche Forschungsgemeinschaft (German Research Foundation)
HYGEDE: Hydraulisch gebundene Deckwerke (hydraulically bound revetments)
IWASA: Internationales Wasserbausymposium Aachen (International Symposium on Hydraulic Engineering, Aachen)
IWW: Institut für Wasserbau und Wasserwirtschaft (Institute of Hydraulic Engineering and Water Resources Management)
MKULNV: Ministerium für Klimaschutz, Umwelt, Landwirtschaft, Natur- und Verbraucherschutz des Landes Nordrhein-Westfalen (Ministry for Climate Protection, Environment, Agriculture, Conservation and Consumer Protection of the State of North Rhine-Westphalia)
